# Canadian adolescents' perspectives of cancer risk: a qualitative study

**DOI:** 10.1093/heapro/dau011

**Published:** 2014-03-17

**Authors:** Roberta L. Woodgate, Jalal Safipour, Ketan Tailor

**Affiliations:** Faculty of Nursing, University of Manitoba, 89 Curry Place, Winnipeg, Manitoba , CanadaR3T 2N2

**Keywords:** adolescent, Canada, children, determinants of health

## Abstract

Research examining adolescents' understandings of cancer and cancer risk is limited. Accordingly, we conducted an ethnographic study that sought to extend our limited understanding of Canadian adolescents' perspectives of cancer and cancer prevention including how adolescents conceptualize and understand cancer risk. This article addresses findings specific to adolescents' perspectives of cancer risk. Seventy-five adolescents (11–19 years old) took part in the study. Two individual open-ended interviews were planned for each adolescent with the second interview occurring 4 to 5 weeks after the first interview. The second interview was complemented by the use of photovoice. Four focus groups, composed of the adolescents who took part in the individual interviews, were also conducted. Data analysis involved both thematic and content analysis. Findings revealed that adolescents conceptualized cancer risk in terms of specific risk factors, with lifestyle factors (e.g., smoking, diet/nutrition and physical inactivity) dominating their discourse. Adolescents rationalized risky health behaviours through use of cognitive strategies that included questioning and evaluating risk information, considering the benefits costs of the cancer risk, and downplaying the impact of the cancer risk. Use of these cognitive strategies helped to make cancer risks more acceptable to adolescents. While adolescents felt that cancer could not always be prevented, they did feel it was possible for individuals to delay getting cancer by lowering the impact of cancer risks through making the right choices. Although more research in this area is needed, the findings from this study may help inform cancer prevention and risk communication programmes and policies.

## INTRODUCTION

Cancer is the world's second leading cause of death after cardiovascular disease (World Health Organization, 2007a,b). Cancer contributes to approximately one in eight deaths globally (World Health Organization, 2007a), although across North America, the figure is close to one in four (American Cancer Society, 2013; Canadian Cancer Society, 2013). In Canada, about two in five Canadians (41% of females and 46% of males) will develop cancer in their lifetime and about one in four Canadians will die of cancer (Canadian Cancer Society, 2013). Each year in Canada, on average, 880 children under the age of 15 years and 412 adolescents are diagnosed with cancer (Public Health Agency of Canada, 2012a,b). In 2009, cancer was the leading cause of disease-related death in Canadian children under the age of 15 years (Canadian Cancer Society, 2013). By and large, there were 2075 new cases per year between 1992 and 2005, and 326 deaths per year between 1991 and 2004 in Canadian youth aged 15–29 years (Canadian Cancer Society Steering Committee: Canadian Cancer Statistics, 2009).

While prenatal and congenital factors are considered risk factors for childhood cancers and environmental risk factors are more often linked to adolescent cancers (Public Health Agency of Canada, 2012a,b), little is known about the causes of cancer among children and adolescents, which makes prevention difficult for this age group (Public Health Agency of Canada, 2012a,b). However, certain behaviours adopted in adolescence and young adulthood, including risky sexual behaviour, alcohol and drug abuse, poor diet and lack of physical activity, are believed to impact cancer risk later in life. This reinforces the need for early intervention through cancer prevention and health promotion programmes for adolescents and young adults (CCS Steering Committee: Canadian Cancer Statistics, 2009). In addition to research that advances our understanding of adolescents' cancer awareness, programmes and interventions that help increase adolescents' knowledge of the contribution of modifiable lifestyle factors to cancer risk are needed (Kyle *et al.*, 2012, 2013).

Current work examining cancer risk in the context of adolescence has been dominated by quantitative studies that give minimal attention to adolescents' experiences and perspectives. However, research that looked at adolescents' own knowledge of cancer and its warning signs revealed that adolescents' recall of cancer warning signs was poor, and approximately one in four adolescents (26.2%) did not know any sign or symptom of cancer. Follow-up work by Kyle *et al.* (Kyle *et al.*, 2013) showed that an educational intervention was an effective way to raise adolescents' awareness of cancer risk factors. Except for the important quantitative research by Kyle *et al.*, (Kyle *et al.*, 2012, 2013), the study of how adolescents perceive cancer and cancer risks in the context of their lives is missing. The majority of research has focused on identifying the type and number of cancer-related risk behaviours of adolescents. The main behaviours examined include poor dietary patterns and physical activity (Plotnikoff *et al.*, 2009; Rutkowski and Connelly, 2011), alcohol use (Shetty and Brown, 2009), ultraviolet (UV) ray exposure (Williams *et al.*, 2011), tobacco use (Kropp and Halpern-Felsher, 2004; Morrell *et al.*, 2010) and sexual activity related to human papillomavirus (HPV) infection, a sexually transmitted virus found to be the primary cause of cervical cancer (Brown *et al.*, 2005; American Cancer Society, 2013). Together, these findings suggest that adolescents are at significant risk of later cancer due to a lack of information about cancer and their lifestyle behaviours.

Lipworth *et al.* (Lipworth *et al.*, 2010) noted that qualitative inquiry could yield rich and nuanced information of how people experience and construct risk in the context of cancer. Currently, we know very little about how adolescents understand cancer and cancer risk, although a better understanding can help inform primary cancer prevention programmes and policies. A few qualitative studies have examined adolescents' perspectives of risk in general. Wall and Olofsson (Wall and Olofsson, 2008), for instance, used cards depicting different types of risk to stimulate risk discussion among Swedish adolescents. They found that adolescents show variation in how they make sense of risk and reported differences in risk perception based on where adolescents resided (i.e. rural/urban setting). Using focus group interviews, Rodham *et al.*, (Rodham *et al.*, 2006) spoke to adolescents about what they considered to be ‘risky behaviours’ in their lives and the factors influencing their participation in these behaviours. Risks involved an uncontrollable outcome associated with potential for harm or negative consequences. Peer group acceptance/non-acceptance and focusing on future success were factors which influenced adolescents engaging in particular risky behaviours. While these risk research studies are informative about adolescents' perspectives of risk, there remains limited work examining adolescents' understandings of risk specific to cancer. Accordingly, we conducted a qualitative study that sought to extend our limited understanding of Canadian adolescents' perspectives of cancer and cancer prevention, including how adolescents conceptualize and understand cancer risk. This article addresses findings specific to adolescents' perspectives of cancer risk.

## THEORETICAL FRAMEWORK

If we hope to develop meaningful and relevant health promotion and cancer prevention programmes for adolescents to reduce their cancer risk behaviours, a solid understanding of their perspectives of cancer and cancer prevention is required. This includes understanding how adolescents conceptualize cancer risk, and how their beliefs steer their behaviours. Belief is considered a central factor for analysing human behaviour in medical sociology (Good, 1994), and is the core concept of health prevention behavioural theories (Becker and Maiman, 1975; Glanz and Rimer, 1995; Bastani *et al.*, 2010; Glanz and Bishop, 2010; Kelly *et al.*, 2011; Gibbons *et al.*, 2012; Radtke *et al.*, 2012). We applied the health belief model (HBM) to explore and help understand how Canadian adolescents conceptualize cancer risk. The HBM is frequently used as the guiding framework in studies with health prevention concern, such as cancer prevention research, where beliefs are more important than overt symptoms (Glanz and Bishop, 2010). While the HBM is composed of a number of key constructs or cognitions, including perceived severity, personal vulnerability, perceived benefits and perceived barriers, cues to action and self-efficacy (Rosenstock, 1966; Rosenstock *et al.*, 1988; Glanz and Rimer, 1995; Glanz and Bishop, 2010; Sharma, 2011; Gibbons *et al.*, 2012), the one that has received the most attention is perceived risk (Gibbons *et al.*, 2012). The HBM theorizes that the way individuals perceive risk and evaluate danger can have an impact on health-related decision-making. Specifically, individuals are more likely to stop an unhealthy behaviour if it is associated with a negative health outcome, that is, the associated risk is perceived to be significant in terms of severity and susceptibility (Rosenstock, 1966; Rosenstock *et al.*, 1988; Glanz and Rimer, 1995; Glanz and Bishop, 2010; Sharma, 2011; Gibbons *et al.*, 2012).

## METHODS

We set out to explore how adolescents perceived cancer and cancer risks, and how these perceptions translated into their daily lifestyles and activities. Exploring the shared understanding and perceptions of adolescents towards cancer and cancer risk lent itself to an ethnographic design using multiple data collection methods. Generally, ethnography involves studying a specific group of people to elicit explanations of their thoughts and beliefs, and how these thoughts and beliefs affect their behaviour (Creswell, 2003).

### Participants and recruitment

A purposive sampling technique was used with the aim to maximize variation in demographic characteristics (e.g., age, gender, SES, urban/rural residency) and cancer experiences (i.e., knowing others with cancer). Information specific to demographic characteristics were obtained through a questionnaire completed by adolescent participants before being interviewed. Recruitment and analysis occurred concurrently with recruitment ending after theoretical saturation was achieved.

### Procedure and data collection

This study took place between December 2007 and October 2010. Three years were necessary in order to achieve the desired sample size and complete data collection and analysis. Additionally, recruitment and data collection had to be intermittently suspended during the schools' holiday and event schedules. Before commencing data collection, we received permission to carry out the study from our university's ethical review board and from the recruitment sites. Parental consent and assent from all adolescent participants was obtained.

Two individual interviews were planned for each adolescent, with the second interview occurring 4 to 5 weeks after the first. Morse and Field (Morse and Field, 1995) reinforce that understanding can only evolve through thick description, and achieving thick description means interacting with and interviewing participants at numerous times. Additionally, more than one interview affords the opportunity for follow-up questions that help clarify or build on issues identified by adolescents during the first interviews. For both interview sessions, the adolescents took part in an open-ended interview that lasted 60–90 min, and was digitally recorded and transcribed verbatim. The open-ended questions and interview technique gave adolescents the opportunity to discuss what they considered important, to have greater control in the interview process and to share information not anticipated by the researcher (Morse and Field, 1995; Prus, 1996; Berg, 2011). For the first interviews, the guide included general questions about cancer, cancer risks and cancer prevention (e.g. How do people get cancer?, What are some risks that teenagers might take that you think might increase their chances of getting cancer?).

Complementing the second interview session was the participatory research method, photovoice. Photovoice is an innovative way for individuals including adolescents to express their understanding and personal meanings of important topics (Harrison, 2002; Strack *et al.*, 2004). The photovoice method was explained to each adolescent participant at the end of the first interview session. Adolescents were given cameras and, over a 4-week period, were asked to take pictures of what they felt depicted cancer, cancer risks and cancer prevention. In the second interview session, adolescents were asked to describe what their photographs meant to them in terms of cancer, cancer risks and cancer prevention (e.g. How does this [theme of the picture as identified by the adolescent] relate to cancer?, Do you think this is a good picture to use in a cancer prevention program … How would you use it?). Fifty-three adolescents (71%) from the first interview session took part in the photovoice method and second interview session. The remaining 22 adolescent (29%) were unable to participate in the photovoice method and second interview session due to scheduling difficulties.

Finally, four focus groups, each consisting of three to four adolescents who were previously interviewed, were conducted to complement existing findings and to gather new group-based knowledge on cancer risks (Morse and Field, 1995; Prus, 1996; Berg, 2011). Field notes describing verbal and nonverbal behaviours of the participants were recorded after each individual and focus group interview.

### Data analysis

Data analysis occurred simultaneously with data collection. The primary analysis applied to develop the themes was consistent with ethnographic methodology. All data emerging from interviews, photographs and field notes informed data analysis specific to themes described in this article. A data management system (QSR International Pty. Ltd. NVivo9.0 for Windows) was used to organize the data. RW was responsible for the development of the thematic framework specific to cancer risk, which was validated by J.S. and K.T. Data were carefully examined and coded for emergent themes. We applied several analytical procedures congruent with ethnography (Roper and Shapira, 2000) including identifying patterns, isolating and organizing domains and seeking attributes for each domain. Relationships and new theme areas among the domains were identified, refined and linked to analytic categories. Discussing initial interpretations with the adolescents themselves during the second interview helped validity measures by supporting emerging themes and revealing new data. In addition to thematic analysis, we used content analysis (Berg, 2011) to identify the number of times adolescents referred to the various risk factors identified in the subtheme, *Cancer risk as ‘risk factors.’* Measures to enhance methodological rigour included prolonged engagement with data, careful line-by-line transcript analysis and detailed memo writing.

## RESULTS

Our sample consisted of 75 adolescents (female: *n* = 53, 73.3%) between 11 and 19 years of age [mean age = 14.5, standard deviation (SD) = 2.1]. While we strived to recruit adolescents from diverse backgrounds, we were unable to achieve adequate diversity in ethnic backgrounds and socioeconomic status. As well, we were unable to achieve equal numbers of male and female adolescents. A summary of the demographic profile of the sample is shown in Table 1.

**Table 1:** Demographic profile of adolescents participants (*N* = 75)

Characteristic	*n*	Percentage
Age group (years)		
11–13	29	38.6
14–16	29	38.6
17–19	17	22.6
Sex		
Male	20	26.6
Female	55	73.3
Ethnicity		
European	47	62.6
Canadian Aboriginal	08	10.6
Other	14	18.6
No response	6.0	08.0
Location		
Urban	42	56.0
Rural	33	44.0
Family history		
Parent with a cancer history	08	10.6
Sibling with a cancer history	03	04.0
Relative with cancer history	11	14.6
No family with a cancer history	53	70.6
Income status		
Low income	06	08.0
Middle income	54	72.0
High income	08	10.6
No response	07	09.3

Three recurrent themes emerged from our study: (a) Perceived risk; (b) Processing cancer risk; and (c) Slowing down cancer. The themes presented are shared collective themes across the participants. The participant quotes are provided as illustrative examples supporting the themes. While there were differences in responses, variation attributable to any of the demographic factors was limited.

### Perceived risk

This theme refers to how adolescents perceived risk in general, and cancer risk in particular. Sub-themes included (a) Risk as taking a chance; (b) Cancer risk as ‘risk factors’; (c) Cancer risk and danger; and (d) Cancer risk and carelessness.

#### Risk as taking a chance

Adolescents equated risk with the concept of taking a chance. Taking a chance involved adolescents doing something out of the ordinary that would result in a change. For example, an 11-year-old female mentioned changing her lifestyle by not following the same routine every day. Risks were a necessary and expected part of daily life, and were not always perceived as something negative:

We're always at risk, walking across the street we can get hit by a car or whatever it is, but I think we definitely need risk and I think that's all a part of having excitement in your life. (15-year-old female, K8)

While partaking in a risk involving uncertainty, adolescents reinforced that by not taking chances, one could miss out on opportunities that may have positive outcomes:

Like taking a risk in going to apply for a job because you don't know what could happen. Like you could get the job, but if you don't apply at all, there's absolutely no chance of getting it. (13-year-old female, C6)

Although adolescents reinforced that risks were a necessary part of everyday life, some risks were more acceptable than others. Risks described as acceptable were risks that adolescents would be willing to participate in and unacceptable risks were risks that adolescents would not part take in. Adolescents did vary on what they defined as acceptable or unacceptable risk, and how much risk they were willing to accept:

It's also okay to take risks, but not huge ones. But like small ones like I'm not one for taking many crazy risks and so like, but just think what could happen… (13-year-old female, C6)

While adolescents varied with respect to whether they viewed a risk to be acceptable or unacceptable, adolescents were more likely to agree that risks to one's health were less acceptable:

Um, I think like sometimes you have to take risks, but with your health you should you should really think about consequences and the actions you are taking.(17-year-old female, K7)

#### Cancer risk as ‘risk factors’

When asked to define the meaning of cancer risk, adolescents provided a definition that consisted of specific risk factors. They identified a number of risk factors including environmental factors (e.g., poor air quality) and biological factors (e.g., sex), although the discourse involving cancer risks was dominated by lifestyle factors or personal habits. In fact, the top five risk factors noted by adolescents pertained solely to lifestyle: cigarette smoking, including second-hand smoke (97.3%), diet/nutrition (60.0%), physical inactivity (46.0%), UV exposure (43.0%) and drug use (31.0%). These trends generally held true regardless of participant age or gender; however, more females (64.0%) identified UV ray exposure as a cancer risk factor compared with males (40.0%), whereas more males (60.0%) identified drug use as a cancer risk factor compared with females (35.0%). Other cancer risk factors mentioned by adolescents were alcohol consumption (33.3%), the absence of early detection (29.3%), genetics (36.0%), age (29.3%) and exposure to chemicals/pollutants (28.0%) and poor air quality (17.3%). Not surprisingly, the majority of photographs also depicted lifestyle factors pertaining to cancer risk (e.g. pictures of fruits, vegetables and exercising). Social (e.g. poverty) and political (e.g. food safety) factors were almost non-existent in the adolescents' discourse and photographs. When political factors were identified, they focused primarily on government smoking policies. Although the lifestyle factors identified above dominated the adolescents' discourse, there were other lifestyle factors such as stress (12.0%) and sexual activity (5.3%) that were mentioned, but rarely discussed or depicted in the photographs. None of the adolescents, even when probed, identified HPV infection as a cancer risk.

All adolescents, regardless of whether they had a history of smoking tobacco, felt that there was a greater chance of getting cancer from tobacco smoke, be it first- or second-hand smoke, compared with partaking in any other cancer risk behaviours:

There is a less chance of getting skin cancer from the sun than there is from second-hand smoke. I think the sun is less of a worry. Smoking is definitely more severe and harder acting. (15-year-old female, K11)

#### Cancer risk and danger

In addition to the type of cancer risk, the risk of cancer was perceived to be more dangerous based on the degree of exposure, which was determined by the amount (e.g., the number of cigarettes smoked in one day) and frequency (e.g., the numbers of days spent smoking cigarettes):

Like tanning to me it, it doesn't seem like a serious issue to me because when I do go I only go in for very short periods of time. I don't go every day for a whole year. I definitely don't do that because I know I've heard far too many stories and things that happen to people that it kind of scares me a bit to go every day. Like I don't know why people would do that. (17-year-old female, R7)

Adolescent participants believed that with an increase in the degree of exposure to cancer risk factors, there was a greater chance of being diagnosed with cancer, and that the cumulative effects of cancer risk factors would affect one's health as an adult:

I think these are things that involve chemicals in cigarettes entering your body and … it's a matter of slowly accumulating like in your body. Same with these chemicals you may be only using so much on your hair today but it's just going in you and going in you … Nobody thinks about it now because you don't see the effects until you're fifty. But ‘no’ we're going to have to start thinking about it now, it's right now and even before that. (17-year-old female, R4)

#### Cancer risk and carelessness

An unhealthy lifestyle and not caring about the consequences further increased the adolescents' perceived risk of getting cancer:

Well I know a major one is smoking … if you're just not taking care of yourself and you're just living in an environment where germs are everywhere … especially it depends on your diet if you're just kind of pigging or whatever and you're just kind of eating food not really caring about (it) … I think people know that they shouldn't be smoking cause that is a big reason why they get cancer so that's a good way to prevent it and then they're kind of being careless. (13-year old female, W8)

Adolescents attributed the notion of carelessness to individuals not caring despite having knowledge of cancer risks, and felt that those partaking in cancer risks especially cigarette smoking, were *‘bringing it (cancer) on’* or *‘looking for it.’* The adolescents often expressed anger or sadness when sharing stories about the recklessness of adults and other adolescents who exposed themselves to the dangers of cancer risks:

It makes me feel sad (in response to a photograph of an adolescent smoking). Just sad because basically he is doing a very slow process of suicide. He is sucking away his life every cigarette he smokes. (14-year-old female, W2)

Participants expressed angry feelings upon sight of people who exposed others to cancer risk:

I'm kind of mad at the actual person who's smoking because they're being selfish and they aren't thinking of the fact that there's someone younger or even older than you who could be in danger. (13-year-old female, W7)

### Processing cancer risk

Processing cancer risk refers to how adolescents evaluate cancer risk factors and rationalize risky health behaviours. Adolescents rationalized risky health behaviours through the use of three cognitive strategies: (a) Cancer risk and managing information; (b) Benefits-costs of the cancer risk; and (c) Downplaying the impact of the cancer risk. The use of these cognitive strategies helped to make cancer risks more acceptable.

#### Cancer risk and managing information

Adolescents managed cancer risk information in different ways, which impacted their perceptions of the dangers of cancer risk. This involved questioning and evaluating risk information in the news, media and from others (i.e., family, friends, rumours), and then determining whether the information was valid, false or inconclusive. In terms of using cell phones, one adolescent expressed the following,

The government needs to stop saying cell phones are causing cancer, breathing is causing cancer, walking is … they need to stop telling us what causes it until they know for a fact … (16-year-old female, R9)

In other instances, adolescents accepted the facts of certain cancer risks, but would then create their own messages that focused on positive aspects of the risk. One adolescent who smoked commented,

I don't tie smoking into cancer at all, like in my head when I picture a cigarette I picture certain things like coffee, I picture things that I relate with cigarettes that are different than things I relate with cancer. That's how I'm able to twist it in my mind. (16-year-old female, R8)

While adolescents were knowledgeable about certain cancer risks, how they managed cancer risk information was influenced by misinformation or misconceptions. This was especially true for adolescents' understanding of UV ray exposure as a cancer risk:

Because you can't always trust a sun-tanning machine, and if you're in the sun you can get like the exact amount of sun that you want, and it is real, not artificial and it is better for your skin. (14-year-old female, E15)

Like why would someone go to a tanning salon when we can just do it like naturally, but I'm guessing that maybe naturally the sun might like do more damage and I'm guessing that a tanning salon probably doesn't? (11-year-old female, C7)

Adolescents also spoke about the lack of information around products or items that may be a potential cancer risk and the work involved in searching out this information:

All these hairsprays they're just like inhaling it, even perfumes I'm sure they're not that great either but you don't know. None of this information is out there really. I mean it is but you, you would have to search hard to figure it out and really make an effort.(14-year-old female, C4)

#### Benefits-costs of the cancer risk

Adolescents considered the benefits to partaking in a risk, and then decided if the benefits outweighed the expected costs. Adolescents would then convince themselves to partake in a risk because the short-term benefits outweighed the long-term negative consequences:

Adolescent: I want to look good now. I want to look tanned and summer's coming and you don't want to have pale skin.

Interviewer: So when would you expect to maybe see some, some negative impact from tanning?

Adolescent: When I'm older you know, like leather skin. (17-year-old female, M9)

Even when adolescents were concerned about the impact of the cancer risk on their health, they nonetheless participated in the cancer risk behaviour because the benefits of the risk helped them in some way. In the words of one male who realized cancer was bad for his health,

Smoking really calms people down so. It really helps me too actually. It helps me not think about it … think about stress and stuff. (17- year-old male, R12)

#### Downplaying the impact of the cancer risk

Adolescents convinced themselves that they were less susceptible to the negative consequences of the cancer risk based on what they considered as risk factors. They felt less vulnerable to the cancer risk by downplaying or lessening their own risk when comparing themselves to others. One adolescent explained why he felt sun tanning was not harmful to him,

I like the colour brown on my skin because girls apparently like it better when I'm brown. Yeah, they like it better and, and it does really look better to me because I don't feel good in white skin. I always have to regulate my skin and I can let my skin in the summer go brown and then in the winter I have to wear all these sweaters and jackets and you don't let the skin come out very often and then it turns white again. So I don't have that much a high percentage of getting skin cancer. (12-year-old male, B8)

In order to feel less susceptible or vulnerable to risk, adolescents would associate their perceived low risk to less exposure and more cautious and careful behaviour:

Well there's one commercial where the guy loses his jaw, another girl has like one of those things in her throat and they're saying how smoking … but it's not going to happen to me cause it says right on the commercials ‘they smoke two packs a day’ but I only smoke twice a day! (17-year-old female, K13)

Adolescents also expressed that while many people engage in risky health behaviours, the risk of cancer diagnosis is not equal for everybody. This was especially the case for smoking tobacco:

Yeah, cause lots of people smoke and then some people get the cancer but then some get lucky and don't get the cancer. (13-year-old female, W11)

### Slowing down cancer

This theme was about the importance of adolescents taking responsibility in making the ‘*right choices*’ to ward off the perceived likelihood of cancer. When asked about ways to prevent cancer, adolescents believed that cancer could not always be avoided:

Because you can't know when you're going to get cancer because cancer is

always in you. You just have to give it a little bit of a push to get it working.(12-year-old male, B8)

Comments such as ‘no one is safe from cancer’ or ‘you can't always prevent it, it just comes’ reinforced that the adolescents felt that everyone was susceptible to cancer risk. However, while adolescents felt that cancer could not always be prevented completely, they did feel it was possible for individuals to delay getting cancer by lowering the impact of cancer risks through making the right choices. The adolescents were definite that what they did to their bodies today would affect them in the future. This included making the right choice to avoid those cancer risks perceived as more harmful. Adolescents also felt that if one was going to partake in risky behaviours, it was important to do so within reasonable limits. This meant minimizing their risk of exposure through restricting the frequency or amount of the risk factor, as well as ensuring that there are appropriate safeguards in place (e.g. applying sunscreen when sun tanning). Most important to minimizing the impacts of cancer risk factors was making choices that helped to build and sustain a healthy body. Adolescents were convinced that a healthy body would not only help counteract the effects of partaking in a risk factor, but would make individuals better prepared to fight cancer if it occurred:

Like, if there is too much fat in your diet you are not getting your right balance and your immune system is probably trying to degrade it or something and then it gets weakened and then a virus could come in or something and then attack you and get cancer. (15-year-old male, W10)

So like if my dad passed his cancer on to me because I have his genes then I might have it. But I think it is like more like your health reasons and if you take care of yourself then it will be like easier, like say if a ten is like being healthy and then cancer is in your genes so then it probably be a seven or an eight, but then not being healthy then it will lead to a four or a five. Like if you do unhealthy things, you'll like be lowered like down to five or six. But then if you're like a healthy person then you're probably be like an eight or nine because you're doing like more like to save yourself. (12-year-old female, C9)

It is worth noting that while adolescents stressed the importance of making the ‘right choices’, they also expressed that they were not always able to do so due to various conditions. For example, adolescents sometimes had difficulty finding places in their community that provide healthy food choices or options for physical activity.

## DISCUSSION

Our study reported on a much under-researched phenomenon, adolescents' understanding of cancer risk. Except for quantitative work by Kyle *et al.* (Kyle *et al.*, 2012, 2013) that detailed adolescents' awareness of cancer and cancer risk factors, the focus has been on the study of specific cancer risk behaviours, such as tobacco smoking (Kropp and Halpren-Felsher, 2004; Morrell *et al.*, 2010), and exposure to UV rays (Sjöberg *et al.*, 2004). To our knowledge, ours is the first qualitative study to examine adolescents' conceptualization of cancer risk.

Our study yielded three main themes. The first theme, *perceived risk*, provided insights around adolescents' conceptualizations of risk and cancer risk. Although adolescents viewed risks as a necessary part of life, they nonetheless viewed risks that negatively impact one's health as less acceptable. While we did not observe or measure adolescents' behaviour, this finding should be viewed as positive in light of the HBM's premise that individuals are more likely not to take part in a behaviour if they view the associated risk of the behaviour as having more severe consequence to one's health (Rosenstock, 1966; Rosenstock *et al.*, 1988; Sharma, 2011).

Our finding, with respect to adolescents' perspectives of cancer risk, revealed that adolescents defined cancer risk in terms of concrete risk factors and, more specifically, in terms of certain lifestyle risk factors (i.e. smoking, diet/nutrition, physical inactivity and UV exposure). Similar to Kyle *et al.*'s (Kyle *et al.*, 2013) study, awareness of smoking, including second-hand smoke as a cancer risk factor was high, whereas awareness of non-modifiable cancer risk factors (e.g. genetics) and other cancer risks (e.g. exposure to pollutants) was low. The focus on lifestyle conditions is not surprising considering the Canadian Institute for Health Information (Canadian Institute for Health Information, 2005) revealed that Canadian adults focus mainly on lifestyle changes to improve health, but rarely consider the socio-political determinants of health. Likewise, lifestyle was the most frequently mentioned source of cancer risk in a textual analysis of the media's presentation of cancer risk in Canadian newspapers (Musso and Wakefield, 2009).

In addition to focusing mainly on lifestyle factors, adolescents also demonstrated confusion or a lack of awareness in their discourse of cancer risk factors. For example, although adolescents identified UV ray exposure as a cancer risk, they were confused about the dangers of UV ray exposure from the sun versus tanning beds. Another example was adolescents' lack of acknowledgement of HPV infection as a cancer risk factor. This finding is in contrast to a study by Kyle *et al.* (Kyle *et al.*, 2013) who reported a third (31%) of adolescents agreed that HPV infection was a known risk factor.

While adolescents lacked understanding around certain cancer risk factors, their discussion revealed two new findings that demonstrated their intelligence and sensitivity in the discussion of cancer risk factors. The first was the finding that revealed that adolescents perceived cancer risk factors to be more dangerous based on the degree of exposure and that cumulative effects of cancer risk factors would affect one's health as an adult, suggesting that youth do think of their future health status. The second finding spoke to the notion of carelessness and how adolescents viewed others as reckless for partaking in cancer risks despite having knowledge of the cancer risks. There was a blame that the victim attitude and strong emotions associated with the notion of carelessness, which suggests risk is not a neutral statistical concept, but instead, signifies danger and emotional threat (Han *et al.*, 2009).

The second theme, *processing cancer risk*, provided insights around how adolescents evaluate cancer risk factors and rationalize risky health behaviours. Although adolescents defined risks to one's health as unacceptable, they were able to justify risky health behaviours by the use of cognitive strategies, similar to strategies used by adults. These strategies include suppressing undesirable information about the riskiness of their behaviour, rationalizing their decision on the grounds that the short-term benefits outweighed long-term dangers and lessening their own risk when comparing themselves to others (Weinstein, 1984; Lipworth *et al.*, 2010). In support of the HBM, the findings revealed that when comparing and evaluating risk factors, adolescents may reduce the perceived severity of the risk, increase the perceived benefits of the risk and consider themselves less vulnerable in order to partake in the risky behaviour (Rosenstock, 1966; Rosenstock *et al.*, 1988; Sharma, 2011; Gibbons *et al.*, 2012). Lipworth *et al.* (Lipworth *et al.*, 2010) contend that the process by which risks are conceptualized is not solely rational or objective, but, in fact, is (re)constructed through a number of cognitive strategies in order for individuals to gain control.

The third theme, *slowing down cancer*, provided insights around adolescents' understanding of how they can reduce the severity of the cancer risk. In preventing cancer, adolescents felt it was important to take responsibility for their health by making the right choices. Congruent with the HBM, adolescents felt it was important to take action against the risk of cancer, but could not always make the right choices due to barriers in their lives (Rosenstock, 1966; Rosenstock *et al.*, 1988; Sharma, 2011; Gibbons *et al.*, 2012).

### Recommendations

Acknowledging further research is needed to confirm and add to the findings, we provide tentative recommendations based on the themes that may be of value to educators and other professionals involved in developing cancer prevention policies and programmes. Overall, the findings reinforce Kyle *et al*.'s (Kyle *et al.*, 2013) acknowledgement of the need for cancer-specific educational interventions to raise adolescents' awareness of cancer risk factors. The adolescents' focus on lifestyle factors, as well as confusion or lack of understanding of certain cancer risk factors, suggest that cancer prevention programmes for adolescents requires a broader conceptualization of cancer risk factors, and strongly point to the need for further research on adolescents' understanding of cancer risk factors beyond diet, exercise and cigarette smoking. Messages and cancer awareness programmes for adolescents should include risk information associated with the amount and frequency of cancer risk, rather than an all or nothing approach (e.g. stay out of the sun). We also suggest that cancer awareness and risk communication programmes and policies not only focus on the *dos* and *don'ts* of cancer prevention, but challenge how adolescents process cancer risks. Case scenarios need to be presented to adolescents that include examples of adolescents managing the information, weighing the benefits-costs and downplaying the impact in the context of cancer risk factors. Likewise, case scenarios depicting examples of adolescents dealing with a variety of barriers that make it difficult for them to take action against the risk of cancer would, be of value. A case scenario approach could be a powerful self-reflective and educative tool to empower adolescents to become more aware of their beliefs and lifestyle practices around cancer risk. Addressing strong emotions associated with cancer risk also needs to be considered when developing cancer awareness programmes. Finally, in addition to the development of cancer-specific educational interventions, research similar to Kyle *et al.* (Kyle *et al.*, 2013), which tests the effectiveness of the interventions, is needed.

### Strengths and limitations

We acknowledge some limitations to this study. Primarily, we did not observe and measure adolescents' behaviour. Adolescents' perspectives of partaking in cancer risk factors may differ from actual behaviour. As well, this was a cross-sectional study and, as such, understanding how perspectives of adolescents change over time was not possible. Aside from the finding that adolescent females and males differed with respect to their perspectives of UV exposure and drugs as cancer risks, we did not detect other differences based on gender or age. This consistency across gender and age could be attributed to the fact that the majority of participants were females (72%), and that only 17% of participants were 17 years of age and older. We did not obtain significant diversity in ethnic backgrounds and socioeconomic status. Future work is called for that accounts for our study's limitations, as it might result in additional perspectives on cancer risks that warrant the attention of cancer prevention efforts and policy development initiatives. A strength of the study was the use of data collection methods including individual open-ended interviews and photovoice, which provided adolescents a powerful way to communicate and share their beliefs, and engage in critical thought and reflection about cancer risk and cancer risk factors.

## CONCLUSION

Research into adolescents' understanding of cancer risk and cancer risk factors is a developing field. Findings from our study afford a deeper appreciation of adolescents' beliefs of cancer risk, as well as how adolescents evaluate cancer risk factors, rationalize risky health behaviours and make the right choices to ward off cancer. Although more research in this area is needed, the findings from this study may help inform cancer prevention and risk communication programmes and policies.

## FUNDING

R.L.W. is supported by a Canadian Institutes of Health Research Applied Chair in Reproductive, Child and Youth Health Services and Policy Research (Grant#: CIHR APR -126339) and was supported by a by a Canadian Institutes of Health Research (Grant#: CIHR MOP - 84398) operating grant for this study. Funding to pay the Open Access publication charges for this article was provided by the Canadian Institutes of Health Research [grant numbers MOP - 84398, APR - 126339 to R.L.W.].
